# LONG-DISTANCE FAMILY CAREGIVERS’ APPRAISAL OF BURDEN AND STRAIN SCALES: FINDINGS FROM THE LDCARE INTERVENTION

**DOI:** 10.1093/geroni/igae098.2417

**Published:** 2024-12-31

**Authors:** Richard Chunga, Verena Cimarolli, Kathrin Boerner, Sara Czaja

**Affiliations:** University of Massachusetts Boston, Boston, Massachusetts, United States; LeadingAge, Washington, District of Columbia, United States; Carl von Ossietzky University of Oldenburg, Oldenburg, Niedersachsen, Germany; Weill Cornell Medicine/Center on Aging and Behavioral Research, New York, New York, United States

## Abstract

Although there is a significant and growing number of long-distance family caregivers (LDCs) of older adults with dementia, most evidence-based interventions have been designed and evaluated for caregivers living geographically close to their care recipients. Commonly used scales were designed for proximate caregivers to assess their levels of burden and strain and used to measure outcomes of caregiver interventions. However, LDCs face unique challenges that such scales may not adequately capture. As part of the feasibility testing of a new, remotely delivered, psychoeducational intervention for LDCs, called LDCare, several commonly used burden and strain scales were administered to 40 participating LDCs. During the pre-intervention assessment, the Zarit Burden Interview-12, Pearlin’s Family Conflict Scale, Job-Caregiving Conflict, and Role Captivity Scale were administered to the LDCs. After each scale, open-ended questions were included to provide an opportunity for the participants to evaluate the scales’ relevance to their experience as LDCs. Across scales, most participants stated that the questions described their thoughts and feelings well in general, however, comments regarding some questions’ irrelevance to long-distance caregiving were most common for the Zarit Burden Interview-12 and the Role Captivity Scale. Participants remarked that lower everyday caregiving engagement and geographic distance rendered some questions difficult to answer. Concerns among some LDCs regarding the scales’ relevance to their situation indicate that further research is needed to better understand how questions may be tailored to their unique experiences.

